# Countdown to 2015: Tracking Maternal and Child Health Intervention Targets Using Lot Quality Assurance Sampling in Bauchi State Nigeria

**DOI:** 10.1371/journal.pone.0129129

**Published:** 2015-06-18

**Authors:** Dele Abegunde, Nosa Orobaton, Habib Sadauki, Amos Bassi, Ibrahim A. Kabo, Masduq Abdulkarim

**Affiliations:** 1 JSI Research & Training Institute, Inc., -Targeted States High Impact Project (TSHIP), Bauchi, Bauchi State, Nigeria; 2 Jhpiego- Targeted States High Impact Project, Bauchi, Bauchi State, Nigeria; University College London, UNITED KINGDOM

## Abstract

**Background:**

Improving maternal and child health remains a top priority in Nigeria’s Bauchi State in the northeastern region where the maternal mortality ratio (MMR) and infant mortality rate (IMR) are as high as 1540 per 100,000 live births and 78 per 1,000 live births respectively. In this study, we used the framework of the continuum of maternal and child care to evaluate the impact of interventions in Bauchi State focused on improved maternal and child health, and to ascertain progress towards the achievement of Millennium Development Goals (MDGs) 4 and 5.

**Methods:**

At baseline (2012) and then at follow-up (2013), we randomly sampled 340 households from 19 random locations in each of the 20 Local Government Areas (LGA) of Bauchi State in Northern Nigeria, using the Lot Quality Assurance Sampling (LQAS) technique. Women residents in the households were interviewed about their own health and that of their children. Estimated LGA coverage of maternal and child health indicators were aggregated across the State. These values were then compared to the national figures, and the differences from 2012 to 2014 were calculated.

**Results:**

For several of the indicators, a modest improvement from baseline was found. However, the indicators in the continuum of care neither reached the national average nor attained the 90% globally recommended coverage level. The majority of the LGA surveyed were classifiable as high priority, thus requiring intensified efforts and programmatic scale up.

**Conclusions:**

Intensive scale-up of programs and interventions is needed in Bauchi State, Northern Nigeria, to accelerate, consolidate and sustain the modest but significant achievements in the continuum of care, if MDGs 4 and 5 are to be achieved by the end of 2015. The intentional focus of LGAs as the unit of intervention ought to be considered a condition precedent for future investments. Priority should be given to the re-allocating resources to program areas and regions where coverage has been low. Finally, systematic considerations need to be given to the design of strategies that address the demand for health services.

## Introduction

The reduction of maternal and child mortalities remain among the top global health priorities particularly in sub-Saharan Africa where the majority of deaths are concentrated. Globally, of the over 7 million deaths in children under-five year that occurred in 2011, at least two-thirds were preventable with simple, low cost interventions [1, 2]. As much as 99% of all global neonatal deaths occur in low- and middle-income countries (LIMIC), mostly in sub-Saharan Africa. [3] With the Countdown to 2015 [4] in view, Nigeria’s 170 million population which represented approximately 2% of the global population accounted for 10.0% of the world's annual maternal and childhood mortality burden [5]. Grouped amongst the countries of Somalia, Mali, DR Congo and Niger, Nigeria was ranked among the 10 “worst places in the world to be a mother” [6]. Despite the high national priority assigned to maternal and child health, which has been mounting for several decades, available evidence suggests that Nigeria is unlikely to achieve the targets in the Millennium Development Goals (MDG) 4 and 5 by 2015 [7, 8]. Nationally, the maternal mortality ratio (MMR) was 545 per 100,000 live births in 2008 [9]. Recent estimates in 2013 of 576 deaths per 100,000 live births [10] was not statistically different from the 2008 estimate. MMRs are about three times higher in the northern region [9]. For example, in 2008 the MMR in Bauchi State in the northeastern region was 1540 per 100,000 live births and 1,500 per 100,000 live births in Sokoto State in the northwestern region. In 2008, only about 8% of births in the northwest region were delivered in health facilities in contrast to the national average of 35% [9]. The northern region as a whole also accounted for 94% of all the national deliveries unassisted with no one present (NOP) [7, 9]. Infant mortality rates are as high as 78 per 1000 live births in Bauchi State and the under-five mortality is 128 per 1000 live births [10]. Nationally, the demand for family planning satisfied is 48.5%. It is considerably lower in the northern regions at 11.9% [10]. Only about 45% of pregnant women had at least four antenatal clinic visits during their pregnancy [9]. Nationally, 39% of deliveries were supervised by a skilled birth attendant; 13% of mothers practised exclusive breast feeding for the first six months, DPT3 coverage rate was 47% and only 23% of eligible children under 5 years received antibiotic treatment for pneumonia [4, 9]. Evidence has shown that neonates were five times more likely to die without a birth attendant, three times more likely to die from births attended by Traditional Birth Attendants (TBA). Births that were delivered outside the health facilities were four times more likely to die [11]. Infants delivered with unskilled birth attendants were four times more likely to develop permanent congenital and early hearing loss (PCEHL) than those delivered with skilled attendance [12]. A 10-year autopsy review in a referral hospital in Cross Rivers State in the southern region found that 43% of maternal deaths occurred at the TBA-managed centres, mostly from preventable causes [13].

In the Countdown to 2015, the United Nations Commission on Information and Accountability for Women’s and Children’s Health proposed a select number of indicators to monitor progress along the continuum of maternal, newborn and child health care [4]. These indicators which span the continuum of care, from pre-pregnancy through pregnancy, birth, postnatal and early childhood include: demand for FP satisfied, at least four ANC visits, skilled attendant at birth, postnatal care for mothers and for babies within two days of birth, exclusive breastfeeding for the first six months of life, three doses of combined diphtheria-tetanus-pertussis (DTP3) immunization coverage and antibiotic treatment for pneumonia. The MDGs have adopted these indicators to monitor progress towards attainment of the recommended three quarters reduction in maternal mortality ratio and childhood mortality rate by two-thirds between 1990 and 2015. The indicators have been used to profile countries’ progress towards the achievement of MDGs 4 and 5 and are considered appropriate for the evaluation of maternal and child health programs and interventions [4]. They are also well suited for the assessment of progress in the improvement of reproductive, maternal, newborn and child healthcare in Bauchi State and in Nigeria. In Bauchi State, such interventions have included the promotion of family planning, antenatal clinic utilization, skilled birth attendant-supervised deliveries, safe cord management practices, postnatal care for mothers and newborns. They also included adequate childhood vaccination, exclusive breastfeeding for at least six months, and access to appropriate treatment of febrile illnesses, particularly malaria and acute pneumonia.

Since 2009, the United States Agency for International Development (USAID) with its flagship program—the Targeted States High Impact Project (TSHIP)—managed by JSI Research and Training Institute, Inc and implemented in two northern Nigerian states (Bauchi and Sokoto), has supported these states to reduce preventable maternal, newborn, infant, and child morbidity and mortality. The project components integrate maternal, newborn and child health with family planning/reproductive health (MNCH/FP/RH) interventions. Its overall goals are to: strengthen state and local government capacity to deliver and promote the use of high-impact MNCH/FP/RH interventions; strengthen the delivery and promotion of high-impact MNCH/FP/RH/ interventions at primary care facilities, and establish essential referral mechanisms; strengthen the roles of households and communities in promotion, practice, and delivery of high-impact MNCH/FP/RH interventions; and improve policies, programming and resource allocation at the state and local levels.

### Objective

The objective of this study was to estimate the impact of the MNCH/FP/RH interventions implemented in Bauchi State in the period from 2012 when interventions were introduced through to 2014, and to evaluate the progress towards the achievement of MDGs 4 and 5 in the Countdown to December 2015.

## Methods and Setting

Bauchi state is located in the northeastern zone of Nigeria with an estimated population of 5.9 million inhabitants projected from 2006 population census. Administratively, it is divided into 20 Local Government Areas (which are similar to provinces). The Lot Quality Assurance Sampling (LQAS) methodology was applied to monitor and evaluate programs and interventions [14, 15] during the course of implementation of TSHIP. LQAS is a small-sample alternative to traditional population based surveys which is increasingly being used for quick and frequent public health evaluations [15]. It is most useful to determine whether or not a “supervision area” (SA) met set program targets or the expected intervention coverage. LQAS facilitates the classification and prioritization of programs or SAs in the course of implementation. Depending on performance relative to expected targets, a program, intervention or SA is either classified as “high priority” if it achieved less than expected levels of performance (relative to predetermined targets or benchmarks) or assigned to “low priority” when expected results have been achieved. This classification schema provides a verifiable basis to shift program resources to lower-performing programs that have potential for higher impact. Estimates from the SAs were typically aggregated to obtain the coverage of an indicator in a given catchment area that was the State in this case.[16, 17]. Baseline and follow-up surveys using LQAS were conducted in November 2012 and March 2014 respectively to monitor impact. In each survey, data were obtained with pretested questionnaires through a rigorous multistage random sampling of 19 LQAS locations (villages and hamlets) in each of Bauchi State’s 20 LGAs. Nineteen households were randomly selected from each of these locations totaling 760 households. Data collection followed an intensive and detailed 9-day training course of data collectors and field supervisors that covered the practical implementation of LQAS, household sampling techniques, data collection, LQAS analysis and utilization of results. Data at the individual and household levels were obtained for 34 indicators including indicators that has been proposed for monitoring countries’ progress towards the MDGs in the continuum of maternal, newborn and child healthcare [4]. The classification of LGA by performance was done using a statistically determined decision rule determined by the SA sample size of 19 households and pre-determined coverage benchmarks (or targets). The LGA estimates from all the 20 LGAs were aggregated and weighted by the proportional contribution of population of each LGA to estimate coverage for the entire State. Confidence intervals were calculated using a finite population correction[18].

### Ethics Statement

Ethical clearance for this study was obtained from the Bauchi state’s Health Research Ethics Review Committee following the submission of the study protocol including the survey questionnaire and the details of the consent procedure and the consent form. Confidentiality policy was explained to all respondents including their rights to decline participations without any risk of any form of retribution, before obtaining informed written consent signed by thumb printing.

### Analysis

Epi Info was utilized for electronic data capture. Descriptive analyses were performed using preprogrammed Microsoft Excel worksheet and STATA Version 10. For the baseline and follow-up surveys, we estimated and compared the average coverage of each indicator for the LGAs with an acceptable level of statistical errors of α ≤ 5%, and β ≤20% [19, 20].

## Results

A total of 860 women of childbearing age (15–49 years old) were interviewed and included in the study; 430 each were interviewed at baseline survey and the follow-up survey. Background characteristics of women in both surveys were comparable. Mean age of the women were 26 years (95% CI: 15–49 years; Standard Deviation (SD): 7 years) and 26.5 years (95% CI: 15–45 years; SD: 7 years)) in the baseline and follow-up surveys respectively. Virtually all of the women interviewed were married (baseline: 99%; follow-up: 98%). Over 93% of the women practice Islam.

**Fig 1:**
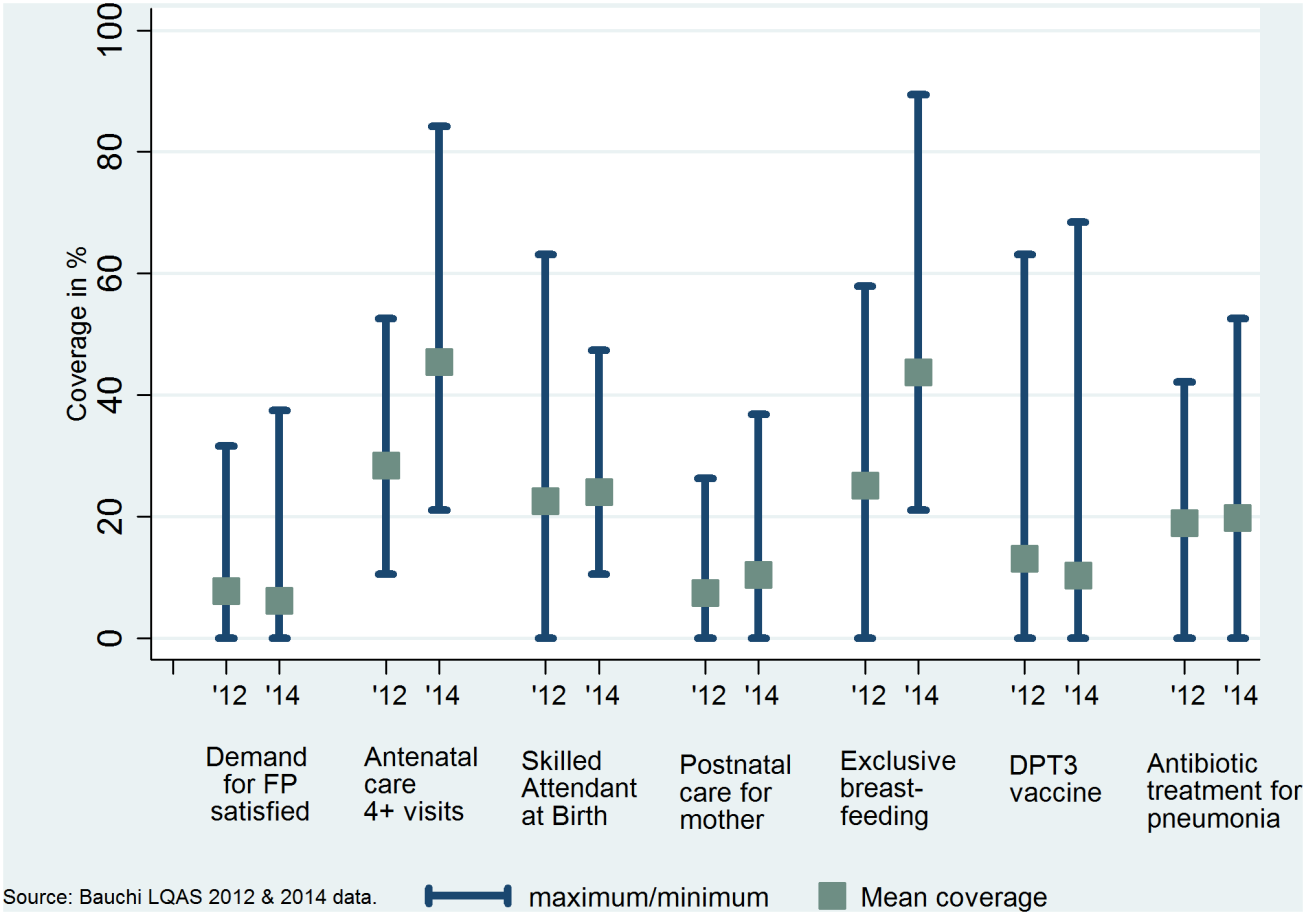
Coverage levels of selected indicators across the continuum of care, 2012 & 2014: Bauchi state coverage range (minimum and maximum) and average.

The coverage estimates of the indicators for the continuum of care at baseline (2012) were all lower than the national-level estimates (obtained from the 2008 and 2013 NDHS [9, 10]) and much lower than the MDG-recommended coverage level of 90% [4] (Table 1). Breastfeeding practices were defined differently in the study and the DHS and the Countdown 2015, and the ensuing estimates were therefore not comparable. The demand for FP satisfied was 7.8% (95% CI: +/- 0.7%) in 2012 compared to follow-up (2014) estimate of 6.1% (95% CI: +/- 1.0%) and the national average of 15%. The demand for FP satisfied had declined by 1.7 percentage points by 2014 from the baseline coverage. Although the estimated coverage of at least four antenatal visits in pregnancy at baseline was also below the 2008 national average of 45%, it increased from a baseline level of 28.4% (95% CI: +/- 1.6%) to 45.5% (95% CI: +/- 2.6%) in the 2014 follow-up survey. Deliveries supervised by a SBA increased by only 1.5 percentage points from the baseline level of 22.5% (95% CI: +/- 1.4%) to 24.0% (95% CI: +/- 1.4%) both of which were lower than the national average of 39%. There was a three-percentage point increase in the percent of women who had received postnatal care within 4 days of delivery from baseline of 7.4% (95% CI: +/- 0.6%) to 10.4% (95% CI: +/- 0.9%) by the follow-up survey in 2014. The coverage of newborns that were breastfed within 8 hours of birth increased by 19 percentage-points from a baseline of 25% (95% CI: +/- 1.6%) to 43.8% (95% CI: +/- 2.5%) in the follow-up. DPT3 vaccination coverage dropped by 2.8 percentage-points from a baseline of 13.1% (95% CI: +/- 1.0%) to 10.3% (95% CI: +/- 0.6%) in the follow-up period. Access to antibiotic treatment for acute pneumonia increased by only one percentage-point from 18.8% (95% CI: +/- 1.0%) at baseline, to 19.8% in the 2014 follow-up. As shown in the range plots in Fig 1, at the aggregate state level, only marginal increases in the average coverage were recorded for most of the indicators in the continuum of care. The exceptions were coverage for four or more antenatal visits and exclusive breastfeeding (in 24 hours before the study) which recorded significant increases. This implies that a few LGAs achieved over 80% coverage in these indicators (Fig 1). We disaggregated coverage by LGAs. In general, the coverage of all the indicators was widely distributed around the average estimates reflecting the large differences between LGAs in the attained coverage estimates. While some LGAs had low coverage rates, others had achieved significantly high coverage at baseline and or follow-up.

**Table 1:** National Averages and Coverage Estimates of the Indicators of the Continuum of Maternal and Child Health Care, 2012 and 2013.

Continuum of care	Indicators	National Estimates—DHS 2008 [9]/(Countdown) [4]	National Estimates DHS 2013[10]	Estimated Average coverage		
				2012. (95% CI)	2014 (95% CI)	Difference: 2013—2012.
**Pre-pregnancy**	Demand for FP satisfied,	15	15	7.8 (0.7)	6.1 (1.0)	-1.7
**Pregnancy**	Antenatal care (4+visits),	45	61^†^	28.4 (1.6)	45.5 (2.6)	17.1
**Birth**	Postnatal care for mother (SBA)	39 (35)	38	22.5 (1.4)	24.0 (1.4)	1.5
**Postnatal Care**	Postnatal care for Mothers			7.4 (0.6)	10.4 (0.9)	3.0
	Post natal care for baby,					
**Childhood**	Exclusive breast feeding: u6m.	13	17	25.14 (1.6)	43.8 (2.5)	18.7
	DPT3 vaccine	35	38	13.1 (1.0)	10.3 (0.6)	-2.8
	Antibiotics for Pneumonia u5	23	29	18.8 (1.0)	19.8 (1.2)	1.0

Estimated Coverage across the continuum of maternal and child health care indicators in percents.

^†^ Proportion of women who reported consulting a skilled health provider—a doctor, nurse, midwife, or auxiliary midwife—at least once for antenatal care for the most recent birth in the five-year period before the survey.

**Fig 2:**
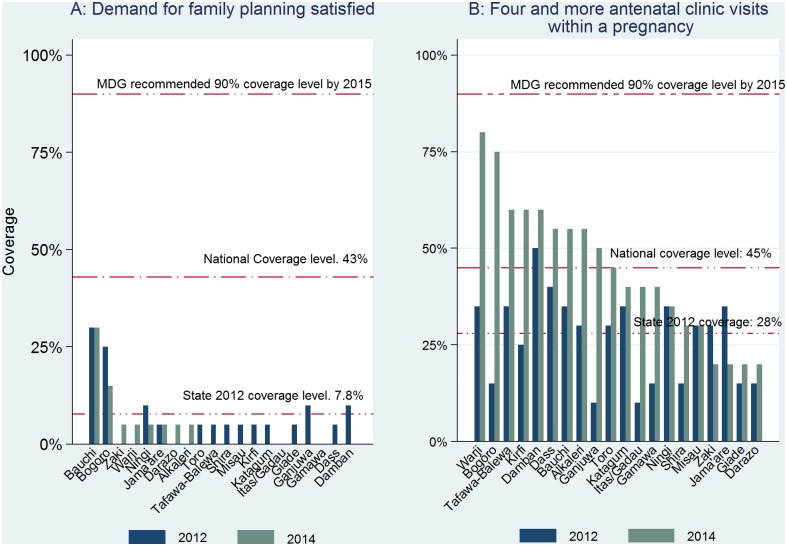
Pre-pregnancy and Pregnancy continuum: coverage levels of demand for family planning satisfied and at least four antenatal clinic visits during a pregnancy by LGA. 2012 and 2013.

Although the estimated average (state) coverage fell short of the MDG recommended and the national coverage for all the Continuum of care indicators in the 2012 baseline and 2014 follow-up, a breakdown of coverage by LGA showed that only a few LGAs achieved very high coverage rates in some of the indicators. Only one of the 20 LGAs (Bauchi LGA) had attained the national coverage level of 43% in the demand for FP satisfied at baseline and follow-up surveys (Fig 2 (A)). The coverage of women who attended at least four ANC visits during pregnancy increased significantly from baseline to follow-up in all the 20 LGAs (Fig 2(B)). None of the 20 LGAs achieved the 90% UN recommended coverage rate; 10 other LGAs had achieved coverage rates above the national average of 45% by the follow-up of 2014. In addition, 16 LGAs had achieved coverage rates greater than the baseline level of 28%.

**Fig 3:**
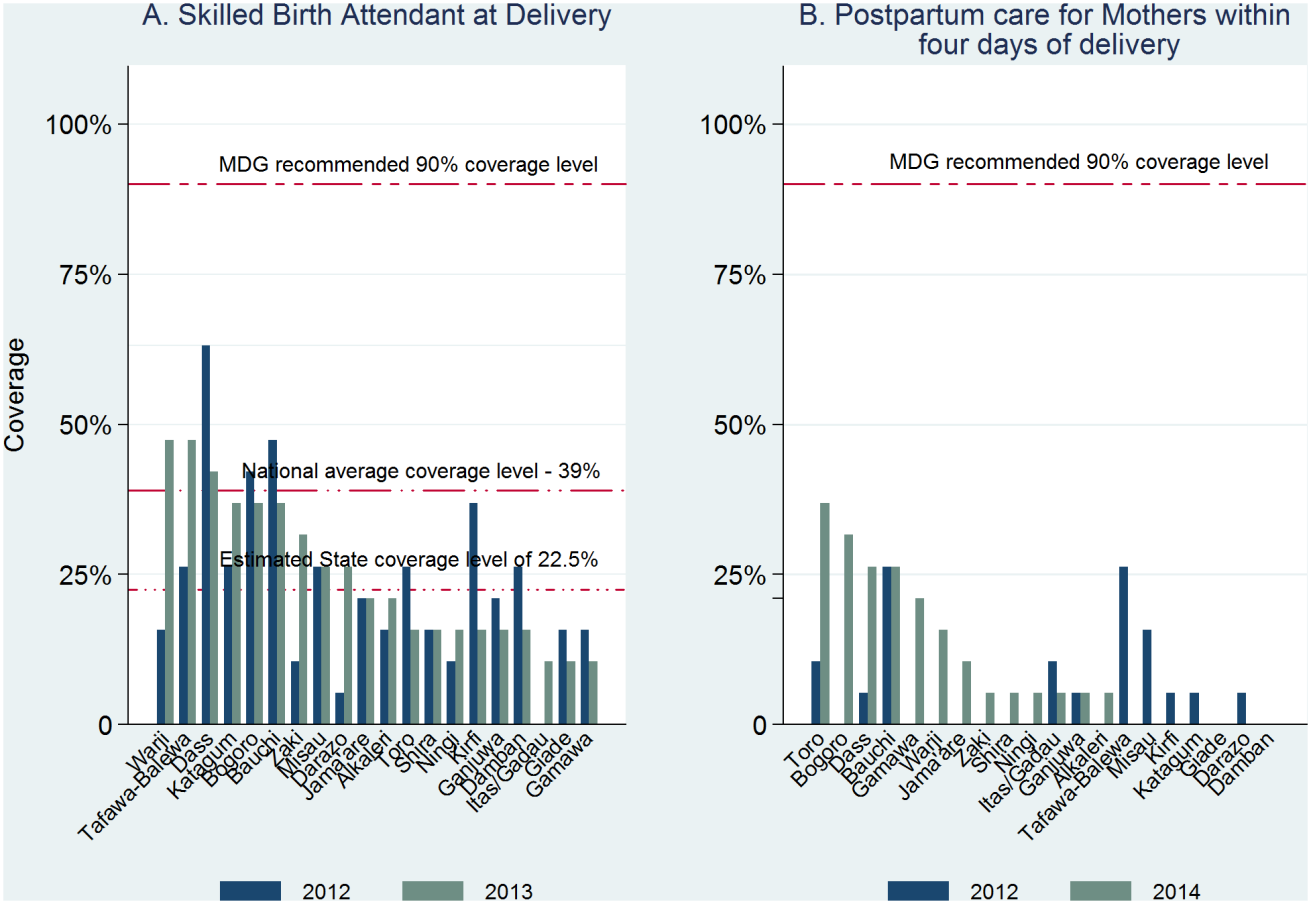
Birth and postpartum continuum: coverage of Skilled birth attendant at delivery and postpartum care at least four days after delivery. 2012 and 2014 by LGA.

**Fig 4:**
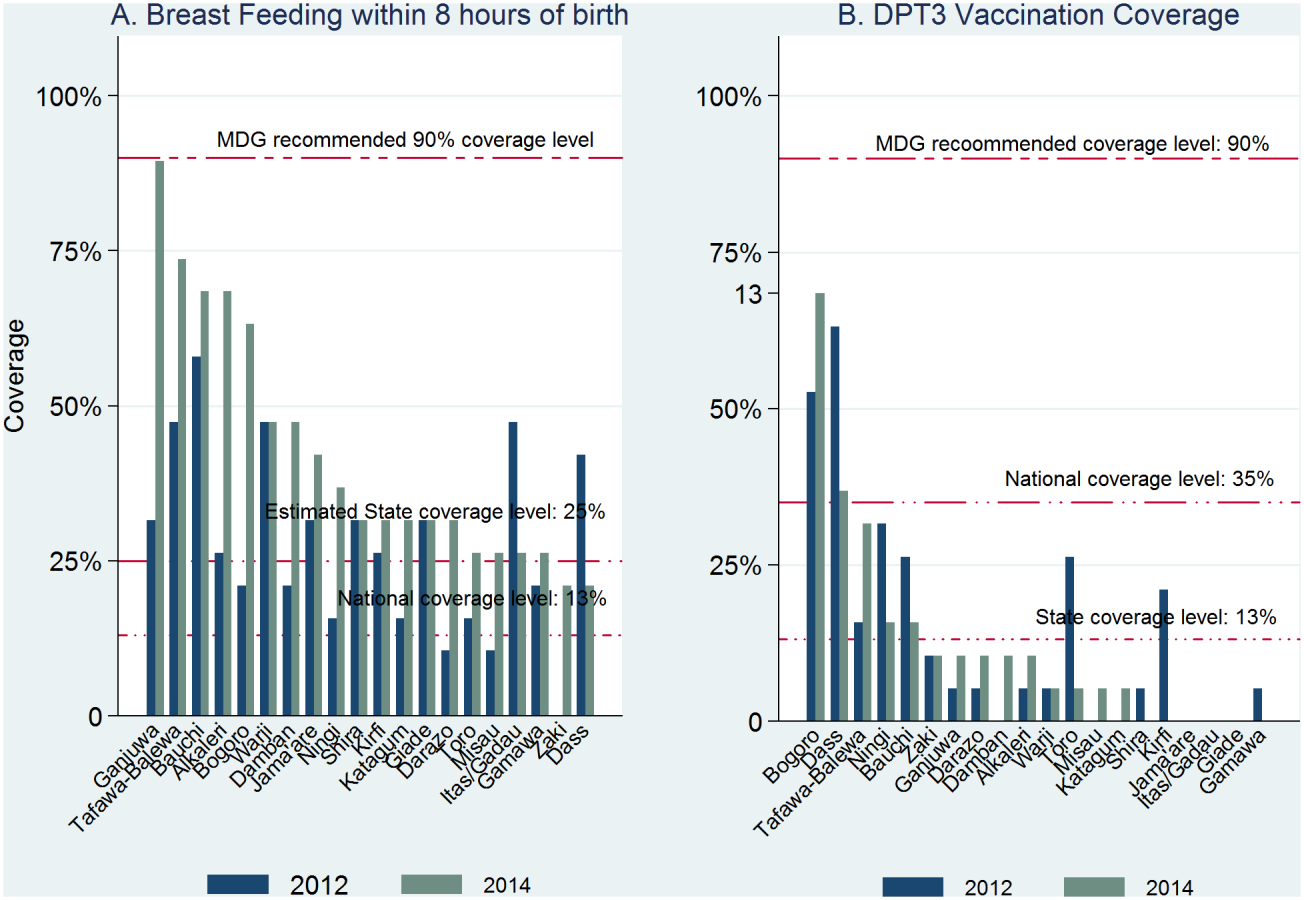
Early childhood: Coverage of breast feeding and DPT3 vaccination by LGA, 2012 and 2014.

None of the 20 LGAs met 90% coverage in the proportion of births supervised by skilled birth attendants at baseline and at follow-up (Fig 3A). However, coverage had increased in 8 LGAs at follow-up over baseline; there was no change in 3 LGAs. In another 9 LGA, SBA coverage had actually dropped by the follow-up. In benchmarking the LGA performances with the national and the estimated State coverage, 3 LGAs had performed above the national average of 39% at the baseline and follow up. In another 6 LGAs, performance levels at follow-up fell between at state average of 22.5% and the national coverage of 39% and 11 LGAs fell below the state average coverage. With respect to the coverage of postpartum care for mothers, while it remained poor at baseline and at follow-up, 10 LGAs recorded a higher percent at follow-up in the number of mothers who received postpartum examination within 4 days after birth. Eight LGAs that had zero coverage at baseline had shown some marginal improvements at follow-up (Fig 3B). The percentage of newborns who were breastfed within 8 hours of birth substantially increased from baseline in 15 of the 20 LGAS, reduced in 2 LGAs, and remained unchanged in 3 LGAs (Fig 4A). One LGA (Ganjuwa) already had a follow-up coverage up to the 90% UN target.

**Fig 5:**
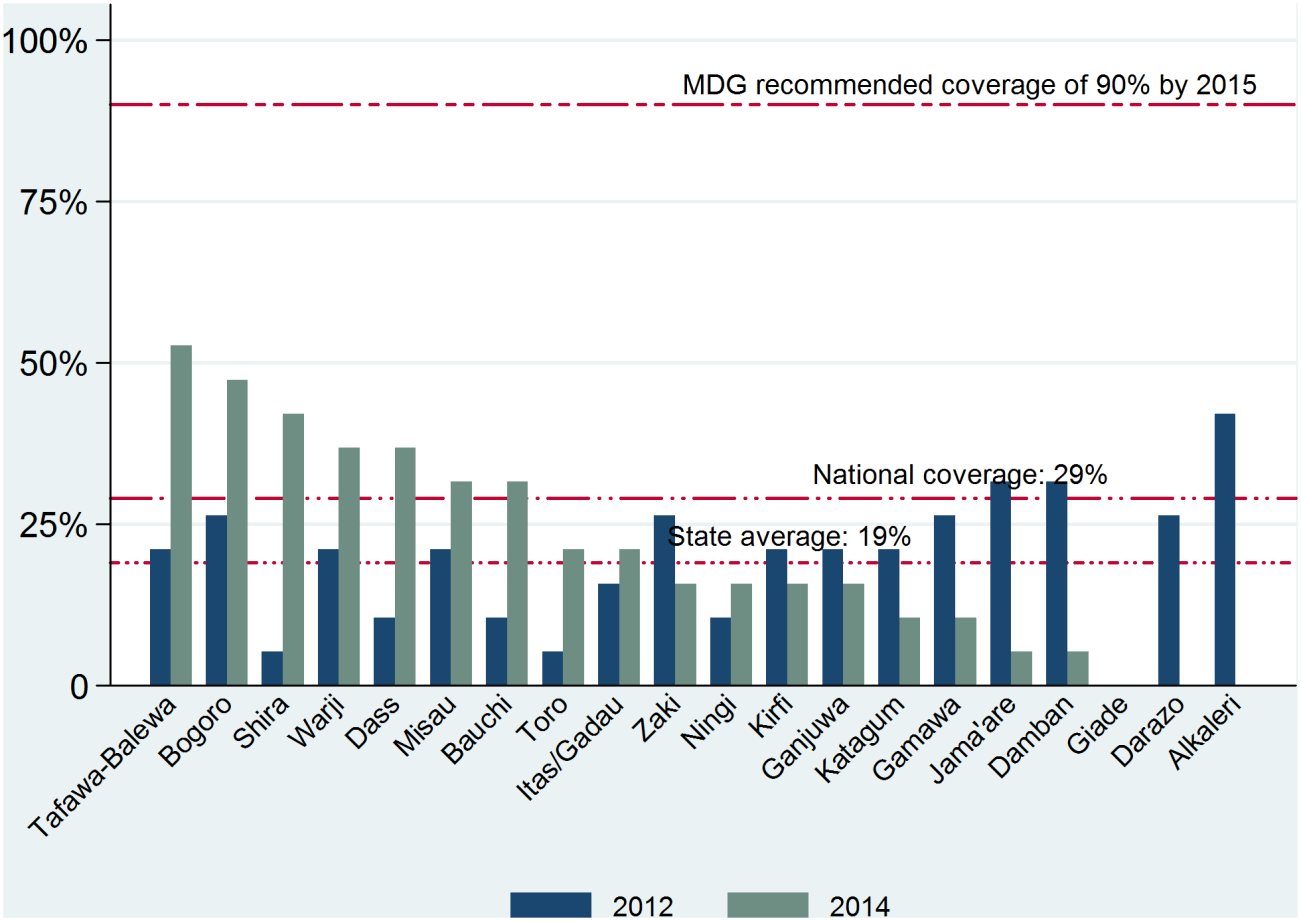
Early childhood: coverage of antibiotic use for pneumonia by LGAs, 2012 and 2014.

The coverage of DPT3 vaccines was below the national coverage of 38% (Fig 4B) in all but two LGAs. There were marginal improvements in the DPT3 coverage in 8 LGAs and 4 LGAs showed a decline in coverage from the baseline. The coverage in the use of antibiotics for acute pneumonia showed slight increase in the follow-up from baseline in 10 of the 20 LGAs, while it dropped at follow-up in 9 LGAs and there was no baseline or follow up coverage in one (Giade) LGA (Fig 5). Although antibiotic coverage estimates exceeded the national average of 29% in only 3 LGAs at the baseline, just 7 LGA had exceeded the national average at follow-up.

## Discussion

Using the framework of indicators proposed by the Countdown to 2015 global monitoring of the continuum of maternal and child health care (from pre-pregnancy to early childhood) [4], this study evaluated the impact of integrated, multipronged interventions designed to improve maternal and child health and the progress towards the attainment of MDG 4 and 5 targets in Bauchi State in northeastern Nigeria. The results were mixed and have implications for reprogramming of interventions in the Countdown to 2015 and for the reduction of maternal and child mortality in Bauchi State and in Nigeria. Throughout the 20 LGAs, relative to baseline, there was a remarkable 18.7 percentage point increase in the percent of newborns that were breastfed within 8 hours of delivery by follow-up. In the same vein, the results also showed a statewide increase in the percent of women who had made at least four ANC visits by follow-up, which translated into a 17 percentage point increase in coverage. Both these interventions relied heavily on the presence of community-based health volunteers (CBHV) that numbered 10 per ward and totaled 3,230 in the entire State. Several of these CBHV actively provided counselling on ANC and early initiation of breastfeeding in newborns. Several of the female CBHV were also likely to be present when child was delivered which afforded them a unique position to assert influence to promote and demonstrate the proper technique of breastfeeding to the new mother. The rise in the coverage of mothers who received postnatal care within four days of delivery and of those mothers whose births were supervised by skilled birth attendants was a more modest 3 percentage points and 1.5 percentage points improvement respectively at follow-up. For DPT3 coverage and the met demand for family planning (proportion of child bearing age women currently using a contraceptive method), reversals in coverage were observed with coverage declines of 2.8 and 1.7 percentage points respectively. At the state level, observed improvements in coverage in indicators across the continuum of care neither attained the national coverage estimates levels from the 2013 Nigerian DHS nor the 90% coverage considered a pre-requisite to achieve a two-thirds reduction in maternal and childhood mortality by 2015.

However, coverage level data disaggregated by LGAs produced considerable variation in the level of progress attained in the indicators across the continuum of care. This variation in magnitude of change in coverage highlight the importance of using LGAs as units of interventions to both deploy interventions as well as in the measurement of change. While a focus on aggregated statewide estimates is useful for between states comparisons, they mask differences between LGAs. For example, four LGAs--Bogoro, Dass, Warji and Bauchi--for the periods under review mostly consistently outperformed the other 16 LGAs and could be classified as positive deviants. Nonetheless, the remaining 16 LGAs fared less well, and recorded worse coverage rates at follow-up, and these would be classifiable as “high priority” according to LQAS benchmarking criterion. As such, these LGAs would be candidates for one or a combination of interventions including program intensification and or a re-appraisal of strategy including the validity of the assumptions in force. It is equally important to dig deeper into understanding the success factors behind high-performing, deviant LGAs to promote cross-learning with lower performing LGAs. For example, the increase in the prevalence of initiation of breastfeeding with 8 hours from 31.5% in 2012 to 89.5% in 2014 in Ganjuwa LGA needs to be critically studied so transferable lessons are spread to other LGAs. Similarly, a critical study of the factors associated with the high DPT3 coverage in Bogoro and Dass LGAs would be highly instructive in rendering guidance to less performing LGAs. We strongly emphasize that further efforts to advance progress in MNCH outcomes now need to consider the state effort, LGA by LGA. In this regard, LQAS is likely to remain versatile and a comparatively cost-effective monitoring tool in the production of disaggregated data by LGA. Its use is likely to grow as an essential requirement for monitoring and tracking progress in MNCH in Nigeria.

A number of factors may explain the modest progress so far. The first is the practice of systematic underfunding of the health system [21, 22]. Only a fraction of budget monies set aside for health is released in practice. Furthermore, the timing of their release is often quite late in the fiscal year to fully leverage the time value of early implementation. Second, this pattern of health financing suggests that the governance mechanisms to assure rational and optimal financing are still nascent. Another critical element is the adequacy of essential human resources to deliver health services[23]. These factors exert considerable influence on the functionality of health centers; non-performing or under-performing facilities are still a significant proportion of the inventory of health facilities in Bauchi State. The marked differences in LGA performance highlight the potential value of increasing the uses of social determinants of health as a framework to refine program and intervention strategies. It is important to consider that the time elapsed between baseline and follow-up may well be too short a gestation period to record larger magnitude of progress. It could also be that some of the more slowly performing areas are more complex in production of change and would require additional time. We also note the interplay of culture, social customs and community perceptions of quality on the demand for health services across the LGAs.

Notwithstanding the modest progress in maternal and child health, these findings have a number of implications to improving maternal and child health in Bauchi State and in the Northern Nigeria to the extent there exist similarities in states’ health systems. For Bauchi State to be on target for the MDG, coverage across the indicators reviewed need to increase by a factor of between three and five. Within the remaining time to December 2015, it is unlikely that these goals will be met without urgent, massive scale-up of programs and interventions, reallocation of resources to priority program areas and to LGAs where coverage has been low.

There were some limitations to this study. First, the short, approximately two-year exposure period between the baseline and follow-up may be too short a period for the detection of sizeable impact. This may account for the marginal increases in coverage found in some of the indicators. A follow-on LQAS at the end of program evaluation in 2015 will help clarify this situation. Second, all responses were retrospectively self-reported by the mothers. We cannot exclude recall bias especially for children of older age. We did however assume that this bias is comparable across the baseline and follow-up surveys. DPT3 was an exception as the responses were verified with vaccination cards. Notwithstanding these limitations, the results compare with similar studies [4, 9, 10] and provided useful information on the progress in maternal and child health.

## Conclusions

Improvements in the continuum of maternal and child health in Bauchi State were significant and modest. Intensive scale-up of programs and interventions is needed in Bauchi State, Northern Nigeria, to accelerate, consolidate and sustain the modest but significant achievements in the continuum of care, if MDGs 4 and 5 are to be achieved by the end of 2015. Insights from the study of positive deviant LGAs and the incorporation of social determinants of heath consideration could yield refined strategies that could better guide efforts to increase program effectiveness for accelerated MNCH outcomes. Guided by these considerations, added gains could be realized through further gains in the enhanced governance for health. The intentional focus of LGAs as the unit of intervention ought to be considered a condition precedent for future investments. Additional funds are likely to be needed. However, their use must be guided by strategic considerations including improved allocative efficiency. Finally, systematic considerations now need to be given to a scrutiny of and the design of strategies that address the demand for health services.
